# 

**Published:** 1897-03-13

**Authors:** 


### EARLY EDITION.

**Notices of Births, Deaths, and Marriages** are inserted in this column at a charge of 2s. 6d. for an announcement not exceeding four lines.

### NOTICE TO CORRESPONDENTS.

All MS., Letters, Books for Review, and other matters intended for the Editor should be addressed The Editor, "The Hospital," The Hospital Building, 28 & 29, Southampton Street, Strand, W.C.

The Editor cannot undertake to return rejected MS., even when accompanied by stamped directed envelope.

**Notice to Advertisers and Subscribers.**

All Advertisements, Orders for Copies of The Hospital, and Business Communications should be addressed to **THE MANAGER, (Not to The Editor),** The Hospital, The Hospital Building, 28 & 29, Southampton Street, Strand, London, W.C.

The Cost of Subscription, post free, is as follows:—Per week, 2½d.; per quarter, 2s. 8d.; per half-year, 5s. 3d.; per annum, 10s. 6d. Foreign:—Per annum, 15s. 2d.; payable, in all cases, in advance.

**NOTICE.—Vol. XX. of "The Hospital" (April to September, 1896), in handsome cloth binding, is now on sale at the Office of this Journal, price 7s. 6d. Cases for binding the Numbers contained in this Volume 1s. 6d. Index to Vol. XX., 2½d., post free.**

**The Monthly Part for March is now ready. Price 6d., by post 8d.**

### BOOKS RECEIVED.

John Wright and Co., Bristol.

"The Medical Annual," 1897.

"Elementary Bandaging and Surgical Dressing." By Walter Pye, F.R.C.S.; revised by G. Bellingham Smith, F.R.C.S.

Religious Tract Society.

"Non-Christian Religions; Their State and Prospect." By the Rev. J. Murray Mitchell, M.A., LL.D.

Whitaker and Sons.

"Whitaker's Directory of Titled Persons."

Cassell and Co.

"Familiar Wild Flowers." Part I. By F. E. Hulme, F.L.S., F.S.A.

Edward Arnold.

"Wasted Records of Disease." By Charles E. Paget.

Bailliere, Tindall, and Cox.

"Quantitative Estimation of Urine." By J. Barlow Smith, L.R.C.P.

J. and A. Churchill.

"Moods: Their Mental and Physical Character."

James Willing.

"Willing's Press Guide."

**Periodicals and Pamphlets.**—Happy Home, Girls' Own Paper, Boys' Own Paper, Sunday Hours, Leisure Hour, Sunday at Home, Westminster Review, London, Windsor Magazine, Cassell's Family Magazine, Literary Digest, Humanitarian.

### SCRAPS AND GLEANINGS.

At the under-mentioned dispensaries the number of patients treated during the past year were as follows: Brighton, Hove, and Preston Dispensary, 15,078 (700 less than in 1895); and Eastern Dispensary, Bath, 4,437 (slightly more than in the previous year).

The Bradford Infirmary Samaritan Society has done good work during the past year, 294 patients having been helped, 118 of whom were sent to convalescent homes. The total number of garments distributed was 957 besides bed linen, and allowances of food, coals, &c.

It is proposed to add a new wing to the Peterborough Infirmary, with the object of providing properly ventilated and detached lavatories, bathrooms, and nurses' kitchens for each of the three wards on the west side of the hospital, and at the same time slightly increasing the size of the wards.

### The Student of Medicine.

### EXAMINATION OF CANDIDATES FOR HER MAJESTY'S ARMY AND INDIAN MEDICAL SERVICES, FEBRUARY, 1896.

The following papers were set:—

Surgery (Sir William MacCormac).

All four questions to be answered.

1. Describe the mode of formation of renal calculus. Mention the varieties met with. What symptoms indicate the presence of stone in the kidney? Give the treatment.
2. What are the causes, and what the diagnosis and treatment of the common form of dislocation of the humerus, both in recent cases and after a considerable interval?
3. Describe the treatment of a compound fracture of the tibia and fibula, and discuss the indications which would determine the necessity for amputation.
4. Give the symptoms, pathology, and treatment of a case of acute glaucoma.

Anatomy and Physiology. (Mr. Makins.)

1. Describe the submaxillary triangle of the neck.
2. Give an account of the arrangement of the synovial membrane of the knee and its extensions. Mention, in their relative positions, the tendons which surround this joint.
3. Describe the form, position, and relations of the supra-renal bodies, and give a short account of what knowledge exists as to their functions.
4. Give a general account of the sources and peculiarities in arrangement of the arterial supply of the brain. Indicate the cortical areas supplied by the anterior, middle, and posterior cerebral arteries respectively.

Chemistry and Materia Medica. (Dr. Shore.)

1. Give an account of phosphorus—(*a*) the state of its occurrence in nature, (*b*) the physical properties of its various forms, (*c*) the formulæ and general properties of its compounds with hydrogen and with chlorine.

What other elements resemble phosphorus in their general chemical properties and in the character of the compounds which they form?

2. Compare the general properties of the "aromatic" or benzine series of organic bodies with the "fatty" or paraffin series.
3. State the chemical composition of chloral and of chloral hydras. How is chloral prepared? State the actions, and mention the chief uses of this substance.
4. Give an account of the effects of overdoses or of too prolonged use f mercurials.
5. Mention the more important drugs which act specially on the spinal cord, indicating, as far as you can, the nature of the action in each case and the particular part of the cord affected.

Natural Sciences. (Dr. Shore.)

[N.B.—Candidates may answer not more than six questions, and they must confine themselves to two branches of science only.]

*Zoology and Comparative Anatomy.*

1. Describe the structural features which characterise the mollusca. Compare the lamellibranchiata with the odontophora.
2. Give an account of the anatomy of the lancelet (amphioxus).
3. Explain the term "alternation of generations," describing the life histories of some organisms which exhibit this phenomenon.

*Botany.*

1. State the morphological characters of the various kinds of bacteria What is "zooglœa" condition?
2. Describe the structure and functions of a typical root.
3. State the diagnostic characters of the following natural orders: rosaceæ, umbelliferæ, coniferæ, orchidaceæ, and gramineæ.

*Physics.*

1. Describe the phenomena called "phosphorescence" and "fluorescence," mentioning substances which exhibit them. Explain the phenomena, as far as you can.
2. Give an account of the circumstances which may affect (*a*) the velocity, (*b*) the intensity of sound.
3. Explain the circumstances which determine the strength of a galvanic current. State Ohm's law. Explain the terms—"Ohm," "Volt," and "Ampère."

*Physical Geography and Geology.*

1. What is the difference between a "volcanic" and a "plutonic" rock? Mention and briefly describe the chief volcanic and plutonic rocks.
2. Describe the production of land and sea breezes. What are the "monsoons"? Explain clearly the terms "cyclone" and "anticyclone."
3. Upon what important factors does the climate of a district depend?

### ROYAL COLLEGE OF SURGEONS IN IRELAND.

Fellowship Examination.—Mr. Ernest George Fenton, L.R.C.S.I., &c., having passed the necessary examination, has been admitted a Fellow of the Royal College of Surgeons in Ireland. The following gentlemen have passed the primary part of the fellowship examination: Mr. F. Warren, L.R.C.S.I., Mr. J. M. M. Crawford, Mr. S. R. Godkin, and Mr. H. Pringle.

### ROYAL NATIONAL PENSION FUND FOR NURSES.

*President*—H.R.H. THE PRINCESS OF WALES.

*Chairman*—WALTER H. BURNS, Esq. *Deputy-Chairman*—HENRY C. BURDETT, Esq

PENSIONS. SICKNESS. ACCIDENT.

### Total Invested Funds, £300,000.

Nurses are invited to join the Fund on account of the substantial and exceptional advantages which it offers them and which *they cannot obtain elsewhere*. The following are the chief points :—

1. **The Fund is Mutual and essentially Co-operative.**
2. **Easy Payment of Premiums.**
   Nur es can pay their premiums monthly or otherwise as best suits their convenience.
3. **The Fund is open to every Nurse.**
   Nurses can assure for Pensions of any amount commencing at any age.
4. **An Investment and Savings Bank.**
   Those entering under the returnable scale can have their premiums returned to them with compound interest, less a small deduction for working expenses. Interest allowed on deposits.
5. **Additions to Pensions.**
   Besides the Pension entered for, substantial additions thereto may be anticipated in the shape of bonuses.
6. **Sickness and Accident Assurance.**
   Policies are issued in connection with Pension policies assuring 10s., 15s., or 20s. a week in cases of incapacity from work through sickness or accident.

**Full particulars to be obtained from THE SECRETARY,**
ROYAL NATIONAL PENSION FUND FOR NURSES,
28, FINSBURY PAVEMENT, LONDON, E.C

